# The Phosphate-Based Composite Materials Filled with Nano-Sized BaTiO_3_ and Fe_3_O_4_: Toward the Unfired Multiferroic Materials

**DOI:** 10.3390/ma14010133

**Published:** 2020-12-30

**Authors:** Artyom Plyushch, Jan Macutkevič, Aliaksei Sokal, Konstantin Lapko, Alexander Kudlash, Dzmitry Adamchuk, Vitaly Ksenevich, Dzmitry Bychanok, Algirdas Selskis, Polina Kuzhir, Juras Banys

**Affiliations:** 1 Faculty of Physics, Vilnius University, Sauletekio 9, LT-10222 Vilnius, Lithuania; jan.macutkevic@gmail.com (J.M.); adamchuk_dzmitry@yahoo.com (D.A.); juras.banys@ff.vu.lt (J.B.); 2 Institute for Nuclear Problems, Belarusian State University, 220006 Minsk, Belarus; bychanok@inp.bsu.by (D.B.); polina.kuzhir@uef.fi (P.K.); 3 Faculty of Chemistry, Belarusian State University, Nezalezhnastsi Ave. 4, 220030 Minsk, Belarus; sokolaa@bsu.by (A.S.); LapkoKN@bsu.by (K.L.); kudlash@bsu.by (A.K.); 4 Faculty of Physics, Belarusian State University, Nezalezhnastsi Ave. 4, 220030 Minsk, Belarus; ksenevich@bsu.by; 5 Radioelectronics Department, Faculty of Radiophysics, Tomsk State University, 36 Lenin Prospekt, 634050 Tomsk, Russia; 6 Center for Physical Sciences and Technology, Sauletekio 3, LT-10257 Vilnius, Lithuania; algirdas.selskis@ftmc.lt; 7 Department of physics and matematics, Institute of Photonics, University of Eastern Finland, Yliopistokatu 7, FI-80101 Joensuu, Finland

**Keywords:** barium titanate, magnetite, phosphates, multiferroic, composite

## Abstract

The composite material filled with nano-sized BaTiO${}_{3}$ and Fe${}_{3}$O${}_{4}$ was designed and studied. The aluminium phosphate ceramics was used as a matrix. The XRD analysis demonstrates only the crystalline structure of the fillers used. The thermogravimetric analysis proves the thermal stability of the composites up to 950 K. The Maxwell–Wagner relaxation was observed in the dielectric spectra of the investigated composites. The dielectric spectroscopy proves the close contact between the nanoparticles with the different ferroic ordering. The phosphate-based composites have been proved to be a prospective candidate for the multiphase multiferroic materials design and development.

## 1. Introduction

The materials, which simultaneously exhibit any of two or more primary ferroic orderings, i.e., ferroelectricity, ferroelasticity or ferromagnetism, are known as multiferroics. The multiferroics recieved a lot of attention due to the perspective to be used as the promissing memory devices: it was expected, that one unit can store four bits of information [1,2]. However, the different ferroic ordering parameters do not act independently, but coupled. That allows to control the magnetic properties by means of the external electric field and vice versa. The single-phase multiferroics are rare, their coupling coefficients are weak and appears at low temperatures [3]. The list of such materials is narrow and includes Gd${}_{2}$CuO${}_{4}$, Sm${}_{2}$CuO${}_{4}$, KNiPO${}_{4}$, LiCoPO${}_{4}$ and BiFeO${}_{3}$. The two-phase composites with ferroelectric (FE) and ferri-/ferromagnetic (FM) phases were proposed as an alternative [4,5]. The coupling coefficient for this materials is achieved by means of the mechanical contact between a piezomagnetic (or magnetostrictive) material and a piezoelectric (or electrostrictive) phases. Such an approach is attractive due to possibility of the independent components selection for the performance at room temperature, huge coupling coefficients [6,7,8] and different connectivities [7,9].

The critical point in these composite materials synthesis is the reactions at the interfaces between the different phases [10]. Usually, the optimization of sintering process should be performed [11,12,13]. As an alternative, the matrix-based composite approach may be proposed. Aluminium phosphate ceramics are the perfect candidate for the role of the matrix: it is chemically and thermally stable, the hardening temperature is relatively low (20–300 ${}^{\circ }$C) [14,15].

However, the phosphate matrix is the complex system, and possible reactions between different phases may occur upon fabrication. The idea of the present research is to study the possibility of the preparation of the multiferroic materials based on the aluminium phosphate matrix. BaTiO${}_{3}$ and Fe${}_{3}$O${}_{4}$ are selected as the functional fillers since they widely studied materials with known electromagnetic properties [16,17,18,19,20]. Such combination of fillers demonstrate advanced dielectric properties [21], coupling coefficient [22] and perspective for the electromagnetic shielding applications [23,24,25]. For the future development of the technology, the main physical properties, i.e., the temperature stability, magnetic properties, and possible electromagnetic features of the inter-phase contacts should be investigated.

## 2. Sample Preparation and Measurement Procedures

Aluminium phosphate ceramic is already complex composite material consisting of binder and filler. The main filler is the mixture of commercially available by Rusal (Moscow, Russia) Al${}_{2}$O${}_{3}$ with average grain size of 1 μm and AlN (average grain size is 60 nm) with mass ratio of 9:1. Aluminium phosphate binder is the interaction product of hydroxide (Al(OH)${}_{3}$) with orthophosphoric acid (H${}_{3}$PO${}_{4}$) with the mole ratio of acid to hydroxide equal to 1:3. The composite ceramic samples were filled with commercially available BaTiO${}_{3}$ (745952, Sigma-Aldrich, Darmstadt, Germany) nanoparticles with average size of 50 nm and Fe${}_{3}$O${}_{4}$ (637106, Sigma-Aldrich, Darmstadt, Germany) with particle size of 50–100 nm.

**Figure 1:**
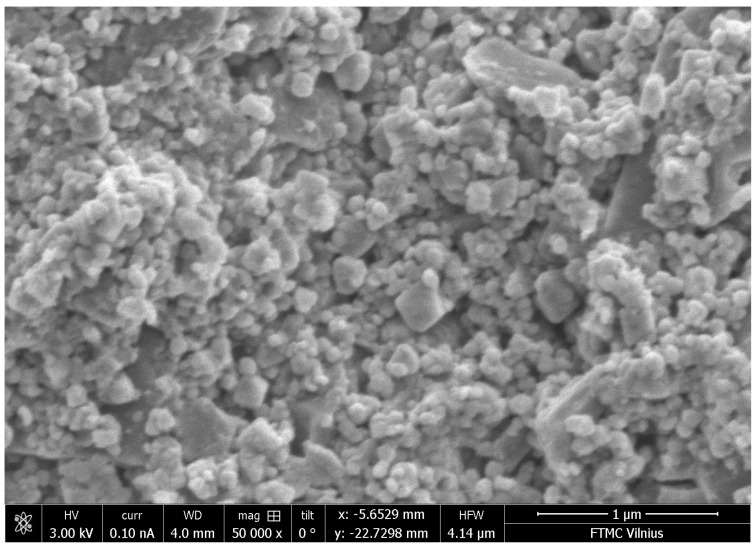
Scanning electron microscopy of the BTFO sample.

The weight concentrations of the components of the samples are presented in Table 1. The filler, the binder and functional fillers were mixed in an agate mortar for 30–40 min. After mixing, the obtained powders were uniaxially pressed under a pressure of 5 MPa, and the tablets were thermally treated up to 600 K for faster curing. The composites filled with BaTiO${}_{3}$, Fe${}_{3}$O${}_{4}$ and hybrid were prepared. The samples are referred as BT, FO for barium titanate and magnetite filled samples respectively and BTFO for hybrid. The distribution of the nanoparticles was controlled by means of the scanning electron microscopy, Helios NanoLab 650 microscope (Thermofisher Scientific, Hillsboro, USA) (Figure 1).

The XRD analysis was performed with DRON-2.0 diffractometer (BOUREVESTNIK, JSC, Saint-Petersburg, Russia), Co Kα (λ = 1.7903 Å) radiation using θ−2θ geometry. The TGA/DGT was done by NETSCH STA 449 (Selb, Germany) with a rate of 10 ${}^{\circ }$C/min in an ambient atmosphere. The samples of the typical mass of 5–10 mg were studied. The magnetization of the Fe${}_{3}$O${}_{4}$-loaded samples was measured using a Liquid Helium Free High Field Measurement System (Cryogenic Ltd., London, UK). The dielectric properties were investigated with an LCR HP4284A meter (Hewlett-Packard, Palo Alto, California). For the measurements, at different temperatures (25–500 K) the closed-cycle cryostat and the home-made furnace was used. The square-like samples with a typical thickness about 1 mm and area of 6 mm${}^{2}$ were investigated. The silver paste was applied for contacting.

## 3. Experimental Results

**Figure 2:**
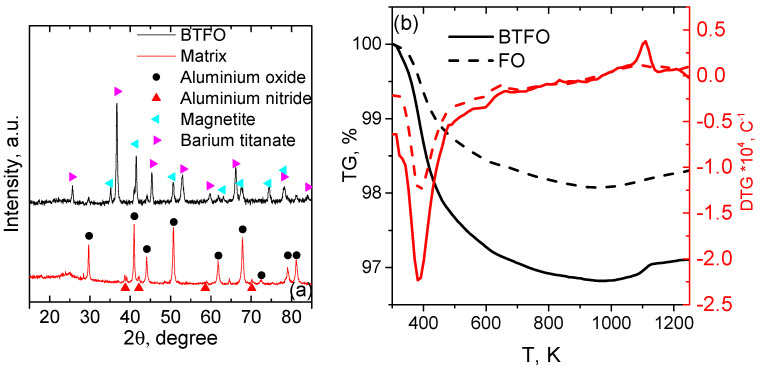
X-ray diffraction pattern of the BTFO and phosphate matrix (**a**). Thermogravimetric analysis of the BTFO and FO samples (**b**).

The comparative X-ray diffraction analysis of the BTFO sample and the phosphate matrix is presented in Figure 2a. The spectrum of the matrix demonstrate only Al${}_{2}$O${}_{3}$ and AlN peaks. The BTFO sample brings the peaks of BaTiO${}_{3}$ and Fe${}_{3}$O${}_{4}$, some of peaks of Al${}_{2}$O${}_{3}$ remains.

The results of the thermogravimetric measurements are presented in Figure 2b. The DTG curves of both samples demonstrate the minimum accompanied by the weight loss of 1–2% at the temperature of 380 K. In the temperature range 480–975 K, a weight loss of 1% is observed due to processes of acid–base interaction and polycondensation of the composite components. At the temperatures of 900–1000 K the weight of both samples starts to increase, that is probably related to the Fe${}_{3}$O${}_{4}$ oxidation [26].

**Figure 3:**
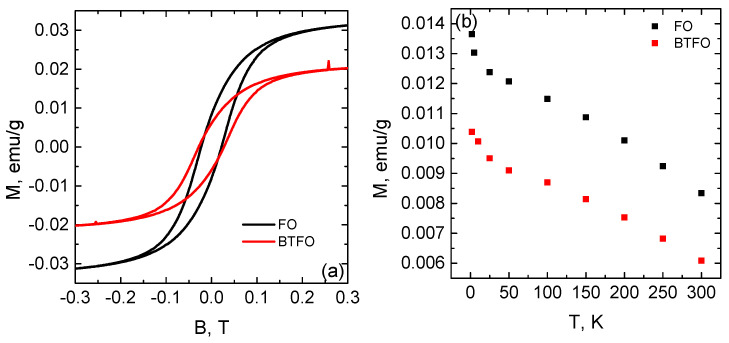
Magnetic hysteresis loops of the BTFO and FO samples (**a**). The temperature dependence of the remnant magnetization (**b**).

The M-B hysteresis loops of the composites are presented in Figure 3a. The hysteresis loops show the presence of the ordered magnetic structure. The reduction of the magnetite phase leads to the decrease of the saturation and the remnant magnetisation. The temperature dependence of the remnant magnetisation (Figure 3b) does not evident the Verwey phase transition due to the size of the Fe${}_{3}$O${}_{4}$ particles [27,28].

**Figure 4:**
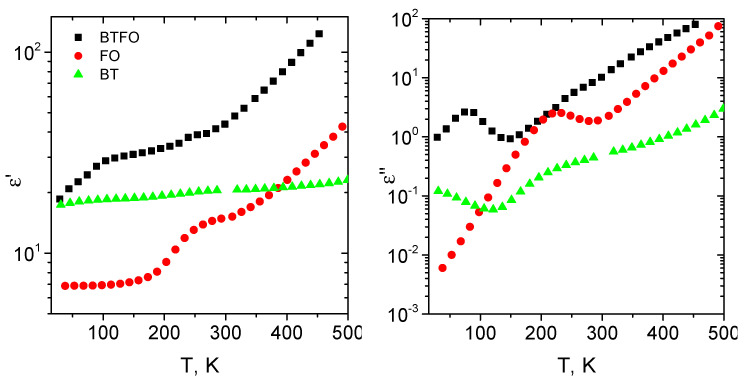
Real and imaginary parts of the dielectric permittivity of the ceramic composites at the frequency of 100 kHz as a function of temperature.

The temperature dependencies of the dielectric properties of the samples are presented in Figure 4. Since the mean particle size of the BaTiO${}_{3}$ particles is lower than the critical size of 110 nm [29], the anomalies related to the phase transitions were not expected in the temperature dependencies [30]. Both parts of the permittivity of the BTFO sample are higher than the ε of the BT or FO samples.

Dielectric properties of BTFO and FO samples are typical for the Maxwell–Wagner relaxation. The relaxation maximums of the dielectric losses of BTFO and FO samples are observed at 75 and 220 K correspondingly. The maximums are followed by dips in $\varepsilon^{\prime }$ dependencies. The behaviour of $\varepsilon^{\prime \prime }$ of the BT sample below 100 K indicates the relaxation maximum below the measurement temperature range.

**Figure 5:**
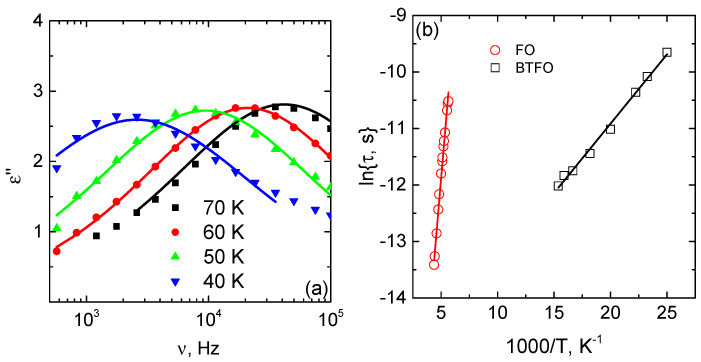
Frequency dependence of the dielectric losses of the BTFO sample: symbols—measured results, curves—Cole–Cole fit (2) (**a**). The temperature dependence of the relaxation time. Symbols—measured results; curves—Arrhenius fit (**b**).

The frequency dependence of the dielectric properties of the inhomogeneous media are simulated using RC-circuit model. The grains and the grain contacts are modelled as the resistor-capacitor parallel unit. The impedance of each unit is $Z=\frac{R}{1+(i\omega \tau )}$, where τ=RC is the relaxation time and ω is the angular frequency. The dielectric permittivity is related to the impedance as:

(1) $$\varepsilon =\frac{1}{i\varepsilon_{0}\omega Z}=\varepsilon_{\infty }+\frac{\varepsilon_{s}-\varepsilon_{\infty }}{1+i\omega \tau }-i\frac{\sigma^{\prime }}{\omega }$$

where τ is the relaxation time, ω is the angular frequency, $\varepsilon_{\infty }$ and $\varepsilon_{s}$—is the high-frequency and static limits of the permittivity correspondingly. The second term describes the ideal Debye-type relaxation. In the studied case, the $\varepsilon^{\prime \prime }$ maximum is broader, and the dependence follows the Cole–Cole law [31] (Figure 5):

(2) $$\varepsilon =\frac{\Delta \varepsilon }{1+{\left(i\omega \tau \right)}^{1-\alpha }}$$

where 0<α≤1 describes the broadness of the relaxation. The temperature dependence of the relaxation time follows the Arrhenius law: $\tau =\tau_{0}exp\left[Ea/kT\right]$. The activation energies for the BTFO and FO samples are 22 and 202 meV, correspondingly.

**Figure 6:**
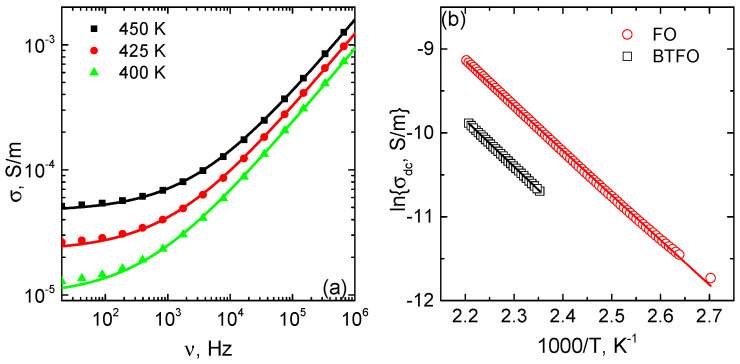
Frequency dependence of the conductivity of the BTFO sample. Symbols are the measured data, curves—fit with Equation (3) (**a**). The temperature dependence of the DC conductivity. Symbols—measured results; curves—Arrhenius fit (**b**).

At higher temperature the BTFO and FO samples demonstrate the frequency-independent plateau of the conductivity (see Figure 6). The frequency dependence of the conductivity is presented as the combination of the dependent and independent terms as [32]:

(3) $$\sigma =\sigma_{DC}+\sigma_{AC}\left(\omega \right)=\sigma_{DC}+A\omega^{r}$$

where $\sigma_{DC}$ is the *DC* conductivity and $A\omega^{r}$ is the *AC* conductivity. The frequency independent $\sigma_{DC}$ depend on the temperature in accordance to the Arrhenius law: $\sigma_{DC}=\sigma_{0}exp\left[-E_{a}^{\sigma }/kT\right]$ (Figure 6). The activation energies of the conductivity are similar for BTFO and FO samples of 0.48 and 0.46 meV, respectively.

## 4. Discussion and Conclusions

The composite materials based on the aluminium phosphate ceramic matrix developed. The nano-sized BaTiO${}_{3}$ and Fe${}_{3}$O${}_{4}$ particles used as the functional fillers. The XRD analysis indicates the presence of the barium titanate, magnetite and matrix peaks (Al${}_{2}$O${}_{3}$ and AlN). The small amount of the amorphous phase is expected as a product of the acid-base reactions [33,34], but in the studied case the halo was not detected. No additional peaks of possible side products were detected. According to TG/DTG analysis, the prepared composite remains chemically stable up to 950 K. At higher temperatures the oxidation of magnetite occurs.

All samples demonstrate the Maxwell-Wagner relaxation behaviour. The phenomenon was studied with RC—circuit modelling. The dielectric permittivity of the composites filled with barium titanate particles depends on the particle size and the concentration of the filler [35,36,37]. The ε of the studied samples is similar to the previously reported data [37]. The hybrid sample has at least twice higher $\varepsilon^{\prime }$ in comparison with BaTiO${}_{3}$ or Fe${}_{3}$O${}_{4}$ filled composite. This is probably related to the additional polarisation on the contacts of BaTiO${}_{3}$ and Fe${}_{3}$O${}_{4}$ grains since FM and FE phases have very different dielectric properties. The intrinsic defects of the nanoparticles even increase the polarization effect [38,39]. The contacts of FM and matrix or FE and matrix does not develop such polarization effects due to very low permittivity and negligibly small dielectric losses of the pure matrix [14]. As a result, the dielectric permittivity and the losses of the BT and FO samples are lower in comparison with BTFO. The difference in the activation energies of the relaxation time supports this idea.

As a result, it was demonstrated, that the phosphate-based ceramic is an attractive matrix for the further multiferroic composite design for several reasons. In contrast to the presented in literature methods of the multiferroic material synthesis [10,11,12,13], the phosphate-based ceramic benefits in the simplicity of the preparation, the ability to avoid the high temperature treatment and the possibility to select the functional fillers independently. In comparison with polymer-based composites [40,41] the inorganic matrix provides advanced thermal stability and mechanical contacts between different phases. Such composites are chemically and thermally stable, the grains of different phases (ferroelectric and ferromagnetic) develop good mechanical contact. The presented methods of the matrix-based composites filled with ferroelectric, ferromagnetic particles and their mixture are promising candidates for the variety of applications. In particular, the composites with ferroelectric nanoparticles are perspective for the memory devices [41,42], electromechanical sensors [40], energy storage [43]. The composites with ferromagnetic particles are applied for electromagnetic shielding [44], construction materials and anti-corrosion coatings [45]. The multiferroic composites are perspective for the range of sensing, transduction and memory applications (see [46] and Refs therein).

## Figures and Tables

**Table 1:** Weight content of the components in the composite materials.

Reference	Main Filler, wt. %	Binder, wt. %	BaTiO${}_{3}$, wt. %	Fe${}_{3}$O${}_{4}$, wt. %
BTFO	26.6	20	26.6	26.6
BT	40	20	40	–
FO	40	20	–	40
